# Use of effective family planning methods and frequency of sex among HIV-infected and HIV-uninfected African women

**DOI:** 10.1186/s40834-018-0063-z

**Published:** 2018-07-09

**Authors:** Jim Aizire, Sufia Dadabhai, Frank Taulo, Bonus Makanani, Luis Gadama, Jin Sun, Amy Tsui, Taha E. Taha

**Affiliations:** 1 Department of Epidemiology, Johns Hopkins Bloomberg School of Public Health, 615 North Wolfe Street, Baltimore, MD 21205 USA; 2 Department of Obstetrics and Gynecology, College of Medicine, University of Malawi, Blantyre, Malawi; 3 Department of Population, Family and Reproductive Health, Johns Hopkins Bloomberg School of Public Health, Baltimore, MD USA

**Keywords:** Contraception, Sexual behavior, HIV/AIDS, Sub-Saharan Africa

## Abstract

**Background:**

Frequency of sex, contraceptive use and HIV infection are key determinants of fertility. Use of an effective family planning (EFP) method (injectable, oral, intra-uterine contraceptive device (IUCD), or Norplant) potentially eliminates women’s concerns of unintended pregnancy. We report the association between EFP and frequency of sex among HIV-infected and HIV-uninfected non-pregnant African women.

**Methods:**

Prospective fertility intentions study nested within a phase 3 randomized double-masked placebo-controlled trial (2003-2005) to treat genital tract infections in HIV-infected and HIV-uninfected non-pregnant women. Enrollment of study participants was stratified by HIV infection status. Data on demographics, family planning and sexual history were obtained at baseline and at 3, 6, 9 and 12 months. Chi square and Wilcoxon Rank-Sum tests were used to compare categorical and continuous variables, respectively. Generalized Estimating Equations method was used to estimate relative risk (RR) of frequent sex (≥ 2 acts/week) among users of different EFP methods (injectable, oral, implant or intra-uterine contraceptive device).

**Results:**

After adjusting for age, current health status, and fertility intentions, EFP use was significantly associated with frequent sex among HIV-infected women (RR 1.32; 95% Confidence Interval [CI] 1.14-1.52); this association was not statistically significant among HIV-uninfected women (RR 1.10; 95% CI 0.96-1.24). Fertility intentions among HIV-infected, and education among HIV-uninfected womenwere independent predictors of sex frequency.

**Conclusion:**

These data suggest that the association between EFP use and frequency of sex among women varies by HIV infection status. Service-delivery of diverse EFP methods should be integrated within HIV counseling, testing and treatment facilities.

**Trial registration:**

Registration number NCT00140764 under the clinicaltrials.gov, first Posted: September 1, 2005, last Update Posted: August 10, 2011.

## Background

Previous studies suggest that the choice of a contraceptive method may positively or negatively influence a woman’s and/or her male partner’s sexual health including sexual desire, arousal and satisfaction [1–6]. Cofactors such as HIV disease are also associated with sexual dysfunction [7–9], and initiation of antiretroviral therapy (ART) has been associated with resumption of sexual libido [9]. However, there appears to be limited data on contraception and sexual health in sub-Saharan Africa, a region with the largest global burden due to unmet contraceptive need and HIV disease, and where scale-up of reproductive health and ART services is ongoing [10–12].

In 2015, with only 10% of the world’s population, sub-Saharan Africa was home to more than 60% of new HIV infections and about 70% of the prevalent cases globally, and approximately 60% of the incident and prevalent cases were reproductive-age women [12]. Only one-in-three of the married women of reproductive age (MWRA) were using some form of contraception, the lowest rate globally [10]. Similarly, the unmet need for family planning (FP) and unintended pregnancy rates are higher in sub-Saharan Africa than in any other region of the world: approximately 19-25% of MWRA had an unmet need for modern or traditional family planning methods [10, 11, 13, 14]; up to 61% of all women regardless of marital status had an unmet need for modern methods [15, 16]; and there were an estimated 108 unintended pregnancies per 1000 women aged 15-44 years [17].

Women are considered to have an unmet need for modern FP methods (sterilization, intrauterine devices (IUD), implants, injectable and oral methods, male condom, vaginal gel and emergency contraception) if they want to avoid pregnancy but are not using any method or are using only traditional methods (withdrawal, rhythm/periodic abstinence, breastfeeding and douching), which are not reliable [10]. Traditional methods are associated with a higher failure rate than modern methods [18]. In sub-Saharan Africa, 87% of unintended pregnancies are attributed to women with a desire to limit or space births who are not using an effective family planning (EFP) method (injectable, oral, intra-uterine contraceptive device (IUCD), or Norplant) [15]. The most commonly reported reasons for not using modern FP by MWRA in sub-Saharan Africa include infrequent sex and safety/side-effects. Others include postpartum/breastfeeding, opposition by the partner, as well as access related reasons [10, 14, 15]. In many countries in the region, one method accounts for 50% or more of all reported FP methods used among MWRA, predominantly injectable (Depo-Provera), a trend attributed to limited varieties of the full range of FP choices and/or unchanged FP messages [10, 19]. In Malawi, the most commonly used methods at the time of this study were injectable contraceptives, and the recent prevalence estimates of the most commonly used methods are: injectable (30%), implants (12%) and 11% female sterilization [20].

Beyond the proximal reproductive health benefits, universal access and utilization of EFP methods has the potential to reduce the high attributable mortality risk among women and children, ameliorate the socioeconomic gender inequities and improve overall health [21–24]. Additionally, EFP scale-up is one of the four prongs adopted by the World Health Organization (WHO) for elimination of mother-to-child transmission of HIV-1 in resource limited settings [25]. The use of EFP methods among women in need of contraception has the potential to increase their self-perceived sub-fecundity and therefore improve their sexual health by disentangling pleasure from the responsibilities of pregnancy.

We report the association between use of EFP methods and frequency of sex among HIV-infected (pre-ART era) and HIV-uninfected post-partum, non-pregnant women in Malawi. In general, while several studies based on African populations previously reported family planning use, the current literature on the specific EFP use and sexual health is limited and no data are available from Malawi. Despite remarkable improvements in the use of modern FP methods (7% (1992); 28% (2004) and 58% (2015-16)), unmet need for FP methods (26% (2010); 19% (2015/6)) and total fertility rate (~ 4.4 children per women) remain high in Malawi [20, 26]. National HIV prevalence was approximaltey 10% in 2010. A national program for the scale-up of ART and injectable contraceptive use among HIV-infected women was implemented in 2011; yet, according to a recent study, the majority (75.0%) of HIV-infected pregnant women on ART reported that their current pregnancy was unintended [27].

## Methods

### Study design and setting

We conducted a prospective cohort study of fertility intentions among HIV-infected and HIV-uninfected women nested within a clinical trial of an intermittent intravaginal antibiotic gel for presumptive treatment of a common genital tract infection - bacterial vaginosis. This trial, known as the METRO study, is registered as trial number NCT00140764 on clinicaltrials.gov. The METRO study was a randomized, double-masked, placebo-controlled, phase 3 trial conducted in Blantyre, Malawi. Overall, 1686 women were enrolled from January 2003 to May 2005: 844 HIV-infected and 842 HIV-uninfected. A detailed description of the study has been previously reported [28, 29]. Briefly, non-pregnant HIV-infected and HIV-uninfected women were recruited via the postnatal care and family planning clinics of the Queen Elizabeth Central Hospital, a tertiary referral hospital in Blantyre, Malawi, and from two nearby health centers. Enrollment of eligible women was stratified by HIV infection status. Women were counseled and provided written informed consent for HIV testing and study enrollment. The criteria for study entry included: age 18 years or older; ability and willingness to give written informed consent; non-pregnant; willingness to return for follow-up visits; willingness to use the intravaginal treatment gel as instructed; willingness to provide specimens at each visit to test for pregnancy and sexually transmitted infections (STIs); and residing in the study area. Exclusion criteria included: inability to provide informed consent; being pregnant; and refusal of any of the enrollment inclusion requirements. The protocol was approved by the College of Medicine Research and Ethics Committee in Malawi and the Johns Hopkins Bloomberg School of Public Health Institutional Review Board in the USA.

### Study procedures

Enrolled women had scheduled visits at enrollment (baseline) and quarterly during follow-up at 3, 6, 9 and 12-months. Demographics, family-planning and sexual history were collected at all study visits using standardized questionnaires by trained study staff. Study participants received standard-of-care counselling on the appropriate family planning methods by trained care providers. Antiretroviral treatment was not available at the time of the study; however, women received standard-of-care such cotrimoxazole prophylaxis through their respective HIV care programs.

### Measurement

#### HIV-1 testing

Women were tested for HIV at trial pre-entry, and at each three-monthly study visit, using a two-tier algorithm including two screening HIV-1 rapid tests or the usual serological tests recommended by the Malawi Ministry of Health. This was followed by a confirmatory Western blot test for those with discordant screening test results and for all new infections.

#### Pregnancy test

Real time pregnancy testing was performed at pre-entry and at each follow-up visit; a detailed analysis has already been published. [29] The detection of the Human chorionic gonadotropin (hCG) hormone in the urine followed by a confirmatory ultra-sound scan was used to diagnose pregnancy. A commercial kit (KAT Quick HCG, KAT Medical, http://www.katmedical.com) which was validated against a gold-standard commercial kit called QuickVue, Quidel Corporation, http://www.quidel.com, was used for urine hCG detection.

#### Exposure – EFP use

The key independent variable was defined as the proportion of women who self-reported using an EFP method which included an injectable, oral, IUCD or Norplant.

#### Outcome - sexual frequency

The outcome in this analysis, sexual frequency, which was assessed at each study visit, was defined as the proportion of women who self-reported 2 or more sexual encounters per week.

### Statistical methods

Data for the METRO trial were entered and cleaned in MS Access. Enrolled women with tubal ligation (*N* = 80) were excluded, while women deaths or losses-to-follow-up, or women with a confirmatory diagnosis of pregnancy during follow-up were censored at that point and therefore did not contribute to this analysis at subsequent follow-up time points. All analyses were conducted separately for the HIV-infected and HIV-uninfected cohorts of women. HIV-uninfected women who seroconverted were classified as HIV-infected at subsequent timepoints. Chi-square and Wilcoxon Rank Sum tests were used to compare categorical and continuous variables, respectively. The Generalized Estimating Equation (GEE) log-binomial model was used to estimate the relative risk (RR) of sex frequency, and the corresponding 95% Confidence Interval (CI),while accounting for within individual correlations from the repeat measures at the multiple visits. To account for key confounding bias, covariates with *p* <  0.15 in the univariate analysis were added to the multivariate GEE model. Fixed variables assessed at baseline included age (in years), education (Form 1-4 and above; Grade 1-8, or no schooling), and number of living children. Time-varying variables included perceived (self-reported) current health status (good/excellent versus poor/fair) and fertility intentions (desire to have another child; not decided/not sure; or no desire to have more children). All statistical tests were two-sided at the alpha = 0.05 level. Statistical analyses were performed using SAS version 9.3, Carey, North Carolina.

## Results

**Fig. 1:**
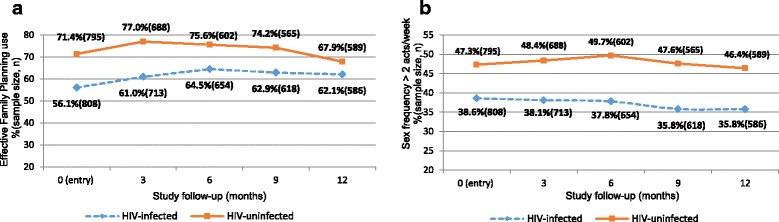
Study visit (0) or entry is the enrollment or baseline visit. Effective Family planning methods which were based on self-reported contraceptive use at study entry and during study follow-up include Injectable, oral, Intra-Uterine Contraceptive Device (IUCD), and Norplant and all the other family planning methods are classified as ‘other’; self-report sexual frequency assessed at each study visit, was defined as the proportion of women who self-reported 2 or more sexual encounters per week. HIV-infection status was based on HIV test results at study entry (enrollment)

The sample sizes of the data available for the baseline and follow-up visit analyses are shown in Fig. 1a and b and Tables 1 and 2. The vast majority of non-pregnant women enrolled in this study were in marital union; 97.3% of the 809 HIV-infected women and 98.5% of the 797 HIV-uninfected women.

**Table 1:** Baseline characteristics of HIV-infected and HIV-uninfected women, Blantyre, Malawi, 2003-2005.

Characteristics	level	HIV-infected (*N* = 809) ^a^	HIV-uninfected(*N* = 797) ^a^	*P*-value ^b^
Age groups, years, n (%)	30+	229 (28.3)	198 (24.8)	*< 0.0001*
	25-29	281 (34.7)	206 (25.8)	
	20-24	271 (33.5)	328 (41.2)	
	< 20	28 (3.5)	65 (8.2)	
Education^c^, n (%)	Form 1-4 or above	241 (29.8)	237 (29.7)	0.91
	Grade 1-8	483 (59.8)	472 (59.2)	
	No Schooling	84 (10.4)	88 (11.0)	
Number of living children, median [IQR]		2 [2-3]	3 [2-4]	*< 0.0001*
Fertility intentions, n (%)	Have another child	215 (26.6)	468 (58.7)	*< 0.0001*
	Not decided/Not sure or No	30 (3.7)	21 (2.6)	
	No more children	564 (69.7)	307 (38.5)	
Family planning methods, n (%)	Effective ^d^	453 (56.0)	568 (71.4)	*< 0.0001*
	No or Other	356 (44.0)	229 (28.6)	
Frequency of sex, n (%)	≤ 2	496 (61.4)	419 (52.7)	*0.0004*
	> 2	312 (38.6)	376 (47.3)	
Treatments, n (%)	Intervention arm	404 (49.9)	399 (50.1)	0.96
	Placebo arm	405 (50.1)	398(49.9)	

^a^Excluded women with tubal ligation (*n* = 80)

^b^*P* values were calculated using Chi-square test for categorical variables, Wilcoxon Rank Sum test for continuous variables. All statistical tests were two-sided at the alpha=0.05 level. Statistically significant differences with a *p*-value equal to or less than 0.05 are italicized

^c^Education (Grade 1-8 of the elementary school; and Form 1-4 of secondary school)

^d^Effective methods include Injectable contraceptives, oral contraceptives, Intra-Uterine Contraceptive Device (IUCD), and norplant; all other methods are classified as ‘others’

**Table 2:** Baseline characteristics of HIV-infected and HIV-uninfected women by family planning method use, Blantyre, Malawi, 2003-2005

		HIV-infected			HIV-uninfected		
Characteristics	Level	EFP^a^ (*N* = 453)	Non-EFP (*N* = 356)	*P*-value^b^	EFP (*N* = 568)	Non-EFP (*N* = 229)	*P*-value
Age groups, *n* (%)	30+	92 (20.3)	137 (38.5)	*< 0.0001*	112 (19.7)	86 (37.6)	*< 0.0001*
	25-29	174 (38.4)	107 (30.1)		147 (26.1)	58 (25.3)	
	20-24	169 (37.3)	102 (28.7)		257 (45.2)	71 (31.0)	
	< 20	18 (4.0)	10 (2.8)		51 (9.0)	14 (6.1)	
Education^c^, *n* (%)	Form 1-4 or above	154 (34.0)	87 (24.5)	*0.014*	179 (31.5)	58 (25.3)	*0.002*
	Grade 1-8	254 (56.1)	229 (64.5)		340 (59.9)	132 (57.6)	
	No Schooling	45 (9.9)	39 (11.0)		49 (8.6)	39 (17.0)	
Number of living children, median [IQR]		2 [2-3]	2 [2-3]	0.34	2 [2-4]	3 [2-4]	0.15
Fertility intentions, *n* (%)	Have another child	108 (23.8)	107 (30.1)	0.12	347 (61.1)	121 (53.1)	0.10
	Not decided/Not sure	16 (3.5)	14 (3.9)		15 (2.6)	6 (2.6)	
	No more children	329 (72.6)	235 (66.0)		206(36.3)	101 (44.3)	
Frequency of sex, *n* (%)	≤ 2	265 (58.5)	231 (65.1)	0.06	299 (52.7)	120 (52.6)	0.98
	> 2	188 (41.5)	124 (34.9)		268 (47.3)	108 (47.4)	
Treatments, *n* (%)	Intervention arm	226 (49.9)	178 (50.0)	0.98	279 (49.1)	120 (52.4)	0.40
	Placebo arm	227 (50.1)	178 (50.0)		289 (50.9)	109 (47.6)	

Excluded women with tubal ligation (*n* = 80)

^a^EFP - Effective methods include Injectable contraceptives, oral contraceptives, Intra-Uterine Contraceptive Device (IUCD), and Norplant; all other methods are classified as ‘others’

^b^*P* values were calculated using Chi-square test for categorical variables, Wilcoxon Rank Sum test for continuous variables. All statistical tests were two-sided at the alpha=0.05 level. Statistically significant *p*-values equal to or less than 0.05 are italicized

^c^Education (Grade 1-8 of the elementary school; and Form 1-4 of secondary school)

At baseline (enrollment) visit, the reported median frequency of sex per week during the past three months was 2 for both HIV-infected and HIV-uninfected women, and mean frequency of sex per week was 2.2 and 2.7, respectively. Among 1130 women with complete data on type of contraceptive method used at baseline, the commonest contraceptive method was the injectable (79.3%) while the frequency of all other methods was low: oral contraceptive pill 8.9%; Implant 1%; IUCD 1%; condoms 4.9%; all others 4.9% (abstinence, withdrawal and breastfeeding). The relative distribution of reported use of contraceptive methods at baseline was similar to and relatively stable during the study period.

Baseline characteristics at enrolment are summarized by HIV infection status in Table 1. Level of education and the treatment arm (MetroGel versus placebo), were comparable between the HIV-infected and HIV-uninfected women (*p* > 0.05). However, HIV-infected women compared to their HIV-uninfected counterparts were older (63.0% vs. 50.6% were 25 years or older, *p* <  0.0001); less likely to report two or more sexual encounters in the previous week (38.6% vs. 47.3%, *p* = 0.0004); and less likely to be using an EFP method (56.0% vs. 71.3%, *p* <  0.0001). In addition, the median number of living children differed by HIV-status of the woman median (IQR) of 2 (2-3) among HIV-infected compared to 3 (2-4) among the HIV-uninfected women, *p* <  0.0001; and less likely to have a desire for another child, 26.6% vs. 58.8%, *p* <  0.0001, respectively.

Differences observed at baseline between HIV-infected and HIV-uninfected women with regard to EFP use and frequency of sex, respectively, were maintained at each of the follow-up visits at 3, 6, 9 and 12-months post enrollment (Fig. 1a and b).

### EFP and sex frequency among HIV-infected women

Baseline characteristics of HIV-infected women by EFP status are summarized in Table 2. Number of living children, fertility intentions, frequency of sex and treatment arm (MetroGel versus placebo), across the EFP use categories were similar (*p* > 0.05). On the other hand, HIV-infected women who reported EFP use versus non-EFP users at baseline were more likely to be younger than 25 years of age (41.3% vs. 31.3%, *p* <  0.0001) and with form 1-4 level of education or higher (34.0% vs. 24.5%, *p* = 0.014).

The following characteristics were associated with frequent sex (two or more times per week) in the bivariate analyses summarized in Table 3: EFP use versus not, RR (95% CI) =1.30 (1.15-1.46); and fertility intentions - desire to have another child versus no desire, RR (95% CI) =1.19 (1.10-1.33). Education, current health status, and number of living children were not associated with sex frequency. In the multivariate model (Table 3) adjusted for age, fertility intentions, and current health status, EFP use versus not was significantly associated with sex frequency, RR (95% CI) =1.32 (1.14-1.52). Compared to women with no desire for children, those with a desire to have another child, RR (95% CI) =1.19 (1.04-1.36), and those not decided, RR(95% CI) = 1.46 (1.12-1.91), were independently associated with sex frequency (Table 3).

**Table 3:** Association of family planning method used and other risk factors with the outcome frequency of sex in HIV-infected women, Generalized Estimating Equations (GEE) Log-binomial Regression Model

Risk factor		Unadjusted RR^a^ (95% CI)^b^	*P*-value^c^	Adjusted RR (95% CI)	*P*-value^c^
Family planning method	Effective	**1.30 (1.15-1.46**)	*<.0001*	**1.32 (1.14-1.52)**	*0.0002*
	No or Other	1	.	1	.
Age (years)	30+	**0.77 (0.55-1.06)**	0.1091	0.95 (0.63-1.44)	0.8200
	25-29	0.99 (0.73-1.36)	0.9741	1.15 (0.78-1.70)	0.4838
	20-24	0.92 (0.67-1.26)	0.5934	0.97 (0.66-1.44)	0.8982
	< 20	1	.	1	.
Education^d^	Form 1-4 or above	1.00 (0.84-1.23)	0.9793		
	Grade 1-8	0.96 (0.79-1.16)	0.6465		
	No Schooling	1	.	1	.
Number of living children		0.98 (0.94-1.03)	0.4894		
Fertility intentions	Have another child	**1.19 (1.10-1.33)**	**0.0033**	**1.19 (1.04-1.36)**	**0.0119**
	Not decided/Not sure	1.26 (0.97-1.63)	0.0852	**1.46 (1.12-1.91)**	**0.0052**
	No more children	1	.	1	.
Current health status	Good or Excellent	1.15 (0.99-1.34)	0.0646	1.12 (0.96-1.30)	0.1492
	Poor or Fair	1	.	1	.

^a^Relative Risk (RR); ^b^Confidence Interval (CI); ^c^*P*-value for test of statistical significance of the RR of sex frequency (bold signifies statistically significant);^d^ Education (Grade 1-8 of the elementary school; and Form 1-4 of secondary school)

Variables with *p* < 0.15 in the univariate analysis were included in the multivariate model

### EFP and sex frequency among HIV-uninfected women

The following baseline characteristics among HIV-uninfected women were homogeneous across the EFP use categories: the number of living children; fertility intentions; frequency of sex; and treatment arm (MetroGel versus placebo), *p* > 0.05 (Table 2). EFP users were more likely to be younger than 25 years of age (54.3% vs. 36.8%, *p* <  0.0001) and with form 1-4 level of education or higher (31.5% vs. 25.3%, *p* = 0.002).

HIV-uninfected women who were using EFP methods compared to those who were not using an EFP method had a 13% higher risk of reporting two or more sexual encounters in the previous week, RR, 95% CI: 1.13(1.01-1.27) (Table 4). However, this association was not statistically significant, OR, 95% CI: 1.10(0.96-1.24) in a multivariate model adjusting for education, number of living children, and fertility intentions(Table 4). A separate multivariate model that included ‘treatment arm’ showed similar estimates or direction of associations. Higher levels of education attained versus no schooling reported at the baseline visit were independently associated with increased likelihood of self-reporting two or more sex encounters in the previous week: Form 1-4 versus no schooling, RR, 95% CI: 1.32 (1.10-1.65); Grade 1-8 versus no schooling, RR, 95% CI: 1.26 (1.04-1.53).

**Table 4:** Association of family planning method used and other risk factors with the outcome frequency of sex in HIV-uninfected women, Generalized Estimating Equations (GEE) Log-binomial Regression Model

Risk factor		Unadjusted RR^a^ (95% CI)^b^	*P*-value^c^	Adjusted RR (95% CI)	*P*-value^c^
Family planning method	Effective	**1.13 (1.01-1.27)**	*0.0356*	1.10 (0.96-1.24)	0.1861
	No or Other	1		1	
Age (years)	30+	1.03 (0.82-1.30)	0.8137		
	25-29	1.18 (0.94-1.48)	0.1533		
	20-24	1.17 (0.94-1.46)	0.1524		
	< 20	1		1	
Education^d^	Form 1-4 or above	**1.36 (1.12-1.65)**	*0.0021*	**1.32 (1.10-1.65)**	*0.0131*
	Grade 1-8	**1.26 (1.04-1.51)**	*0.0156*	**1.26 (1.04-1.53)**	*0.0173*
	No Schooling	1		1	
Number of living children		0.97 (0.94-1.01)	0.1390	1.00 (0.96-1.04)	0.9830
Fertility intentions	Have another child	**1.12 (1.01-1.24)**	*0.0251*	1.10 (0.97-1.22)	0.1695
	Not decided/Not sure	1.17 (0.87-1.57)	0.2976	1.20 (0.89-1.63)	0.2333
	No more children	1		1	
Current health status	Good or Excellent	1.08 (0.92-1.26)	0.3525		
	Poor or Fair	1			

^a^Relative Risk (RR); ^b^Confidence Interval (CI);^c^*P*-value for test of statistical significance of the RR of sex frequency (bold signifies statistically significant); ^d^ Education (Grade 1-8 of the elementary school; and Form 1-4 of secondary school)

Variables with *p* < 0.15 in the univariate analysis were included in the multivariate model

## Discussion

We assessed the temporal relationship between EFP use and frequency of sex among HIV-infected and HIV-uninfected reproductive-age African women followed for 12 months. Family planning use and other socio-behavioral data were collected prospectively every three months as a part of a large randomized placebo-controlled trial for treatment of a common genital tract infection. There was a statistically significant association between EFP use and increased frequency of sex among HIV-infected women after controlling for potential confounders including age, fertility intentions, and current health status. Among HIV-uninfected women, the adjusted association between EFP use and frequency of sex, which was not statistically significant, was in the expected, same direction. Other factors independently and significantly (*p* <  0.05 after adjusting for other variables) associated with frequency of sex in this cohort of non-pregnant women who are in union included fertility intentions among HIV-infected women, and level of education among HIV-uninfected women. The differences observed in this study based on HIV infection status may reveal the complex biological and behavioral factors that impact the sex behavior and fertility decisions of women in sub-Saharan Africa.

The positive association between EFP use and sexual activity in this analysis is consistent with overall findings from a cross-sectional study that analyzed Demographic and Health Survey (DHS) data of more than 210,000 sexually active women of childbearing age from 47 countries suggesting that couples using contraception compared to non-users were three times more likely to report frequent sex [30]. The DHS data, however, was not stratified by HIV infection status of the woman. Other studies, mostly among non-African populations, that evaluated sexual activity and use of family planning methods [2, 4, 6, 31] tended to have more educated women, and were less likely to have high background biological cofactors and socio-economic and cultural constraints that might influence women’s sexual decisions. Women who are more educated and/or reside in settings with less inhibition to openly discuss sex issues may actively consider use of different types of EFP methods to achieve their desires and expectations. Additionally, it is likely that the ability to conceive is valued differently in different settings. For example, in western countries fertility is very low and influenced by economic factors related to the cost of raising and educating a child. In African countries where HIV prevalence is high among women of reproductive age, the impact of HIV on survival of children and their parents is a major determinant of fertility. Successful treatment of HIV with antiretroviral drugs may reverse this health situation and reduce anxieties attributable to concerns of potential vertical transmission in the event of an unintended pregnancy and/or sexual transmission to their HIV-uninfected partners [31].

The heterogeneity of the EFP use and sexual activity measures of association by HIV status observed in this study, is consistent with previous reports of psychosocial, behavioral and biological differences pertaining to sexual function between HIV-infected versus uninfected individuals. A trend towards decreased sexual activity and fertility was observed with advancing HIV disease among Ugandan women in a clinic-based prospective cohort study [7] and a longitudinal study in France showed that the proportion of women who self-reported being sexually active decreased after they discovered that they were HIV-infected [8]. Also, during the convalescent phase after initiating ART, HIV-infected individuals have shown improved sexual desire and resumption of / increased sexual activity [9]. This study predated the introduction of universal ART for all HIV-infected individuals in Malawi, and none of the study women reported ART use. This study enabled a unique opportunity to better understand the sexual benefits of EFP use among HIV-infected women in the absence of ART exposure. In the current era of ART scale-up, an estimated half a million (67%) of HIV-infected individuals and more than 37,000 (73.0%) of the pregnant or breastfeeding women in Malawi were alive on ART through the end of 2014 [26]. This trend towards scale-up of universal ART is consistent across the high HIV burden countries in sub-Saharan Africa, albeit at different paces [12].

At the time the study was conducted, highly sexually active women may have been more motivated to seek and adhere to family planning services including ongoing counseling and utilization of EFP methods. In sub-Saharan Africa, about one in five married women with an unmet need for contraception reported that they were reluctant to use modern contraception methods because of their self-perceived sub-fecundity given their low level of sexual activity [14, 15].

The study was adequately powered (≥ 80.0%) to detect differences in frequent sex among EFP versus non-EFP users, separately for HIV-infected and HIV-uninfected women, respectively. However, the reported measures of association may be biased due to residual-confounding from unmeasured factors, of which intimate-partner violence (IPV) is noteworthy. Previous IPV studies conducted in similar African settings reported a gender-based hierarchy that undermines women’s ability to negotiate condom use or refusal to have sex [32]. Similarly, IPV has been associated with unintended pregnancy and HIV acquisition among women [32, 33]. Also, socio-economic dependence on the male partner is pervasive in sub-Saharan African settings and may be a hindrance to women’s utilization of reproductive health services and likewise increase their vulnerability to imbalanced sexual relationships [9, 32, 33].

## Conclusion

These study results can be generalized to reproductive-age women in need of contraception in other settings in Malawi and elsewhere in southern Africa with similarly high levels of unmet need for EFP, high HIV burden, and where reproductive health and ART services are being scaled-up. The findings are timely and underscore the call-to-action by the WHO and other stakeholders of an integrated approach for the ongoing country level rapid scale-up of ART and reproductive health services in Africa [11, 25, 31]. Women’s sexual health issues will be front-and-center in their decision-making about choice and adherence to family planning methods (in parallel, HIV-infected women are regularly counseled about adherence to their ART use). Avenues should be explored to address women’s sex health issues while integrating the counselling messages on family planning and ART during follow-up. It is critical for adherence to family planning that women make a choice for contraception that is effective, convenient and safe.
